# Acute Hepatitis and Adenovirus Infection Among Children — Alabama, October 2021–February 2022

**DOI:** 10.15585/mmwr.mm7118e1

**Published:** 2022-05-06

**Authors:** Julia M. Baker, Markus Buchfellner, William Britt, Veronica Sanchez, Jennifer L. Potter, L. Amanda Ingram, Henry Shiau, Luz Helena Gutierrez Sanchez, Stephanie Saaybi, David Kelly, Xiaoyan Lu, Everardo M. Vega, Stephanie Ayers-Millsap, Wesley G. Willeford, Negar Rassaei, Hannah Bullock, Sarah Reagan-Steiner, Ali Martin, Elizabeth A. Moulton, Daryl M. Lamson, Kirsten St. George, Umesh D. Parashar, Aron J. Hall, Adam MacNeil, Jacqueline E. Tate, Hannah L. Kirking

**Affiliations:** ^1^Division of Viral Diseases, National Center for Immunization and Respiratory Diseases, CDC; ^2^Epidemic Intelligence Service, CDC; ^3^Department of Pediatrics, Division of Pediatric Infectious Diseases, University of Alabama at Birmingham, Birmingham, Alabama; ^4^Alabama Department of Public Health; ^5^Department of Pediatrics, Division of Pediatric Gastroenterology, Hepatology, and Nutrition, University of Alabama at Birmingham, Birmingham, Alabama; ^6^Children’s of Alabama, Birmingham, Alabama; ^7^Department of Pathology, University of Alabama at Birmingham, Birmingham, Alabama; ^8^Jefferson County Department of Health, Birmingham, Alabama; ^9^Division of High-Consequence Pathogens and Pathology, National Center for Emerging and Zoonotic Infectious Diseases, CDC; ^10^Synergy America, Inc., Duluth, Georgia; ^11^Department of Pediatrics, Division of Pediatric Infectious Diseases, Baylor College of Medicine, Houston, Texas; ^12^Texas Children’s Hospital, Houston, Texas; ^13^Wadsworth Center, New York State Department of Health; ^14^Department of Biomedical Sciences, University at Albany, Albany, New York.

During October–November 2021, clinicians at a children’s hospital in Alabama identified five pediatric patients with severe hepatitis and adenovirus viremia upon admission. In November 2021, hospital clinicians, the Alabama Department of Public Health, the Jefferson County Department of Health, and CDC began an investigation. This activity was reviewed by CDC and conducted consistent with applicable federal law and CDC policy.*

Clinical records from the hospital were reviewed to identify patients seen on or after October 1, 2021, with hepatitis and an adenovirus infection, detected via real-time polymerase chain reaction (PCR) testing on whole blood specimens, and no other known cause for hepatitis. An additional four children were identified, for a total of nine patients with hepatitis of unknown etiology and concomitant adenovirus infection during October 2021–February 2022. On February 1, 2022, a statewide health advisory^†^ was disseminated to aid in the identification of cases at other facilities in Alabama; no additional patients were identified.

All nine children were patients at Children’s of Alabama. These patients were from geographically distinct parts of the state; no epidemiologic links among patients were identified. The median age at admission was 2 years, 11 months (IQR = 1 year, 8 months to 5 years, 9 months) and seven patients were female (Table). All patients were immunocompetent with no clinically significant medical comorbidities.

**TABLE:** Demographics, clinical characteristics, laboratory testing results, and clinical outcomes in a cluster of pediatric patients with acute hepatitis and adenovirus infection (N = 9) — Alabama, October 2021–February 2022

Demographic	No.
**Age at admission, yrs**	
0–2	5
3–4	1
5–6	3
**Sex**	
Female	7
Male	2
**Race**	
White	9
Other	0
**Ethnicity**	
Hispanic	6
Non-Hispanic	3
**Initial sign/symptom**	
Vomiting	7
Diarrhea	6
Fever	5
Upper respiratory symptoms*	3
**Initial physical exam**	
Scleral icterus	8
Hepatomegaly	7
Jaundice	6
Hepatic encephalopathy	1
Splenomegaly	1
Ascites	0
**Liver function testing on admission, median (range)^†^**	
ALT (U/L)	1,724 (603–4,696)
AST (U/L)	1,963 (447–4,000)
Total bilirubin (mg/dL)	7 (0.23–13.5)
**Pathogen testing performed**	
Blood viral PCR	9
Hepatitis A/B/C	9
Epstein-Barr Virus, blood viral PCR	9
Epstein-Barr Virus, IgM	8
Respiratory panel testing^§^	8
Blood culture	4
Urine culture	4
Stool culture	1
**Pathogen testing result, no. positive/total no. **	
Adenovirus (whole blood)	9/9
EBV^¶^	6/9
Enterovirus/Rhinovirus	4/8
Metapneumovirus	1/8
Respiratory syncytial virus	1/8
Human coronavirus OC43	1/8
SARS-CoV-2**	0/9
Hepatitis A/B/C	0/9
**Outcome**	
Recovered without transplant	7
Required transplant and recovered	2
Died	0

**Abbreviations:** ALT = alanine aminotransferase; AST = aspartate aminotransferase; EBV = Epstein-Barr virus; IgM = immunoglobulin M; PCR = polymerase chain reaction.

* Upper respiratory symptoms were identified when taking the patient’s history and conducting an initial physical exam. Upper respiratory symptoms can include nasal congestion, nasal discharge, cough, sore throat, wheezing, and dyspnea, among other symptoms.

^†^ Normal ranges are ALT = 9–25 U/L; AST = 21–44 U/L; total bilirubin = 0.1–1.0 mg/dL.

^§^ The respiratory viral panels (ePlex Respiratory Pathogen Panel [GenMark] or BioFire Respiratory Panel [Biomérieux]) were used to test for adenovirus, coronavirus 229E, coronavirus HKU1, coronavirus NL63, coronavirus OC43, human metapneumovirus, human rhinovirus/enterovirus, influenza A, influenza A/H1, influenza A/H1–2009, influenza A/H3, influenza B, parainfluenza 1, parainfluenza 2, parainfluenza 3, parainfluenza 4, respiratory syncytial virus A, respiratory syncytial virus B, *Chlamydia pneumoniae, Mycoplasma pneumoniae, Bordetella parapertussis* (BioFire only), and *Bordetella pertussis* (BioFire only).

^¶^ Positive EBV test results were based on PCR testing, but all patients received negative test results for EBV IgM antibodies (except one patient who did not have IgM testing) suggesting that infections were likely not acute but rather potential low-level reactivation of previous infections.

** All patients received testing for SARS-CoV-2 using nucleic acid amplification tests.

Before admission, among the nine patients, vomiting, diarrhea, and upper respiratory symptoms were reported by seven, six, and three patients, respectively. At admission, eight patients had scleral icterus, seven had hepatomegaly, six had jaundice, and one had encephalopathy (Table). Elevated transaminases were detected among all patients^§^ (alanine aminotransferase [ALT] range = 603–4,696 U/L; aspartate aminotransferase [AST] range = 447–4,000 U/L); total bilirubin ranged from normal to elevated (range = 0.23–13.5 mg/dL, elevated in eight patients). All patients received negative test results for hepatitis viruses A, B, and C, and several other causes of pediatric hepatitis and infections were ruled out including autoimmune hepatitis, Wilson disease, bacteremia, urinary tract infections, and SARS-CoV-2 infection. None of the children had documented history of previous SARS-CoV-2 infection.

Adenovirus was detected in whole blood specimens from all patients by real-time PCR testing (initial viral load range = 991–70,680 copies/mL). Hexon gene hypervariable region sequencing was performed on specimens from five patients, and adenovirus type 41 was detected in all five specimens. Low viral loads precluded sequencing among three patients, and residual specimens were not available for sequencing for one patient. Seven patients were coinfected with other viral pathogens (Table). Six received positive test results for Epstein-Barr virus (EBV) by PCR testing but negative test results for EBV immunoglobulin M (IgM) antibodies (one patient did not have IgM testing), suggesting that these were likely not acute infections but rather low-level reactivation of previous infections. Other detected viruses included enterovirus/rhinovirus, metapneumovirus, respiratory syncytial virus, and human coronavirus OC43. 

Liver biopsies from six patients demonstrated various degrees of hepatitis with no viral inclusions observed, no immunohistochemical evidence of adenovirus, or no viral particles identified by electron microscopy. Three patients developed acute liver failure, two of whom were treated with cidofovir (off-label use) and steroids, and were transferred to a different medical facility where they underwent liver transplantation. Plasma specimens from these two patients were negative for adenovirus by real-time PCR testing upon arrival at the receiving medical facility, but both patients received positive test results when retested by the same real-time PCR test using a whole blood specimen. All patients have recovered or are recovering, including the two transplant recipients.

Adenovirus type 41 is primarily spread via the fecal-oral route and predominantly affects the gut. It is a common cause of pediatric acute gastroenteritis typically with diarrhea, vomiting and fever, often accompanied by respiratory symptoms (*1*). Adenovirus is recognized as a cause of hepatitis among immunocompromised children (*2*). It might be an underrecognized contributor to liver injury among healthy children (*3*); however, the magnitude of this relationship remains under investigation.

This cluster, along with recently identified possible cases in Europe (*4*–*6*), suggests that adenovirus should be considered in the differential diagnosis of acute hepatitis of unknown etiology among children. Clinicians and laboratorians should be aware of possible differences in adenovirus test sensitivity for different specimen types; tests using whole blood might be more sensitive than those using plasma. CDC is monitoring the situation closely to understand the possible cause of illness and identify potential efforts to prevent or mitigate illness. Enhanced surveillance is underway in coordination with jurisdictional public health partners. Clinicians are encouraged to report possible cases of pediatric hepatitis with unknown etiology occurring on or after October 1, 2021, to public health authorities for further investigation.^¶^

## Footnotes

* 45 C.F.R. part 46.102(l)(2), 21 C.F.R. part 56; 42 U.S.C. Sect. 241(d); 5 U.S.C. Sect. 552a; 44 U.S.C. Sect. 3501 et seq.

†
https://www.alabamapublichealth.gov/bcd/assets/adph_han_report_adenovirus_020122.pdf

§ Normal ranges are ALT = 9–25 U/L; AST = 21–44 U/L; total bilirubin = 0.1–1.0 mg/dL.

¶
https://emergency.cdc.gov/han/2022/pdf/CDC_HAN_462.pdf

## References

[1] Kang G. Viral diarrhea. In: Quah SR, ed. International encyclopedia of public health. 2nd ed. Cambridge, MA: Elsevier; 2017:360–7. https://www.sciencedirect.com/referencework/9780128037089/international-encyclopedia-of-public-health

[2] Hierholzer JC. Adenoviruses in the immunocompromised host. Clin Microbiol Rev 1992;5:262–74. 10.1128/CMR.5.3.262 1323383 PMC358244

[3] Munoz FM, Piedra PA, Demmler GJ. Disseminated adenovirus disease in immunocompromised and immunocompetent children. Clin Infect Dis 1998;27:1194–200. 10.1086/514978 9827268

[4] UK Health Security Agency. Increase in acute hepatitis cases of unknown aetiology in children. London, United Kingdom: Department of Health and Social Care, UK Health Security Agency; 2022. https://www.gov.uk/government/publications/hepatitis-increase-in-acute-cases-of-unknown-aetiology-in-children/increase-in-acute-hepatitis-cases-of-unknown-aetiology-in-children

[5] Marsh K, Tayler R, Pollock L, Investigation into cases of hepatitis of unknown aetiology among young children, Scotland, 1 January 2022 to 12 April 2022. Euro Surveill 2022;27. 10.2807/1560-7917.ES.2022.27.15.2200318 35426362 PMC9012090

[6] World Health Organization. Multi-Country – acute, severe hepatitis of unknown origin in children. Geneva, Switzerland: World Health Organization; 2022. Accessed April 23, 2022. https://www.who.int/emergencies/disease-outbreak-news/item/multi-country-acute-severe-hepatitis-of-unknown-origin-in-children

